# Metaplastic breast carcinoma with osseous differentiation: A report of a rare case and literature review

**DOI:** 10.1515/biol-2022-0640

**Published:** 2023-07-24

**Authors:** Cong Huang, Haibo Tian, Jinming Xu, Fuyun Tong, Dengyang Fang

**Affiliations:** Department of General Surgery, Chongqing University Fuling Hospital, Chongqing, 408000, P. R. China; Chongqing Institute of Minimally Invasive Surgery, Chongqing University Fuling Hospital, Chongqing, 408000, P. R. China; Department of Pathology, Chongqing University Fuling Hospital, Chongqing, 408000, P. R. China; Department of Thyroid and Breast Surgery, Chongqing University Fuling Hospital, Chongqing, 408000, P. R. China

**Keywords:** metaplastic breast carcinoma, breast oncology, breast invasive carcinoma, osteosarcoma, thoracic oncology

## Abstract

Metaplastic matrix-producing breast carcinoma is a type of metaplastic breast carcinoma (MBC), which is a rare malignancy, accounting for 0.2–1% of breast carcinomas. A 52-year-old female visited a hospital because of a palpable painless mass in the right breast and was diagnosed with Breast Imaging Reporting and Data System (BI-RADS) category 4A via ultrasound (US) with a suspected positive lymph node at the right axillary region. Excision of the breast mass was performed and histopathologically confirmed that it was MBC with osseous differentiation. No distant metastasis was revealed before a modified radical mastectomy; however, metastasis to a lymph node of the right axillary region was observed (1/22). She received six cycles of TEC scheme chemotherapy (docetaxel, epirubicin, and cyclophosphamide, 21 days) and 5 weeks of radiotherapy (48 Gy/25 f/5 days a week), but without any follow-up examinations since radiotherapy. Twenty-four months after surgery, distant metastases to lungs and liver were confirmed and died 3 months later. This case provides valuable information for clinicians on MBC and suggests that further examination or biopsy should be performed to US BI-RADS 4A masses before surgery. In addition, regular postoperative follow-up plays important roles in detecting metastases early and improving prognosis.

## Graphical abstract

**Figure:**
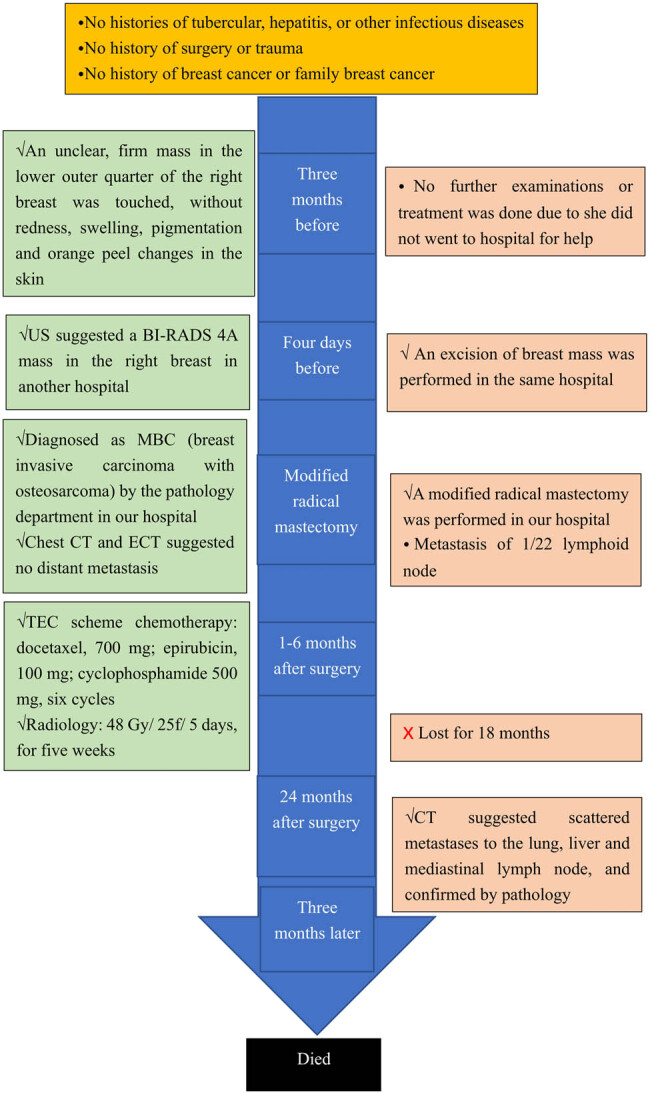

## 1 Introduction

Metaplastic breast carcinoma (MBC) is a group of heterogeneous invasive carcinomas characterized by the differentiation of tumor cells into spindle and/or mesenchymal cells [1], accounting for 0.2–1% of breast carcinomas [2]. It was first described as a mammary carcinoma with mixed epithelial and sarcomatoid components in 1973 [3]. The etiology and pathogenesis of MBC remain unclear [4]. This heterogeneous group of MBC can be histologically classified into the following six distinct groups based on the World Health Organization 2019 classification of breast tumors: (i) low-grade adenosquamous carcinoma, (ii) fibromatosis-like metaplastic carcinoma, (iii) spindle cell carcinoma, (iv) squamous cell carcinoma, (v) metaplastic carcinoma with heterologous mesenchymal differentiation (including chondroid, osseous, and other types of mesenchymal differentiation), and (vi) mixed metaplastic carcinoma [1]. MBC with osseous differentiation is rare with great challenges in diagnosis, treatment, and prognosis. Here, we report the case of a 52-year-old female diagnosed with MBC with osseous differentiation, without any history of carcinoma or family history of breast cancer.

## 2 Case presentation

**Figure 1:**
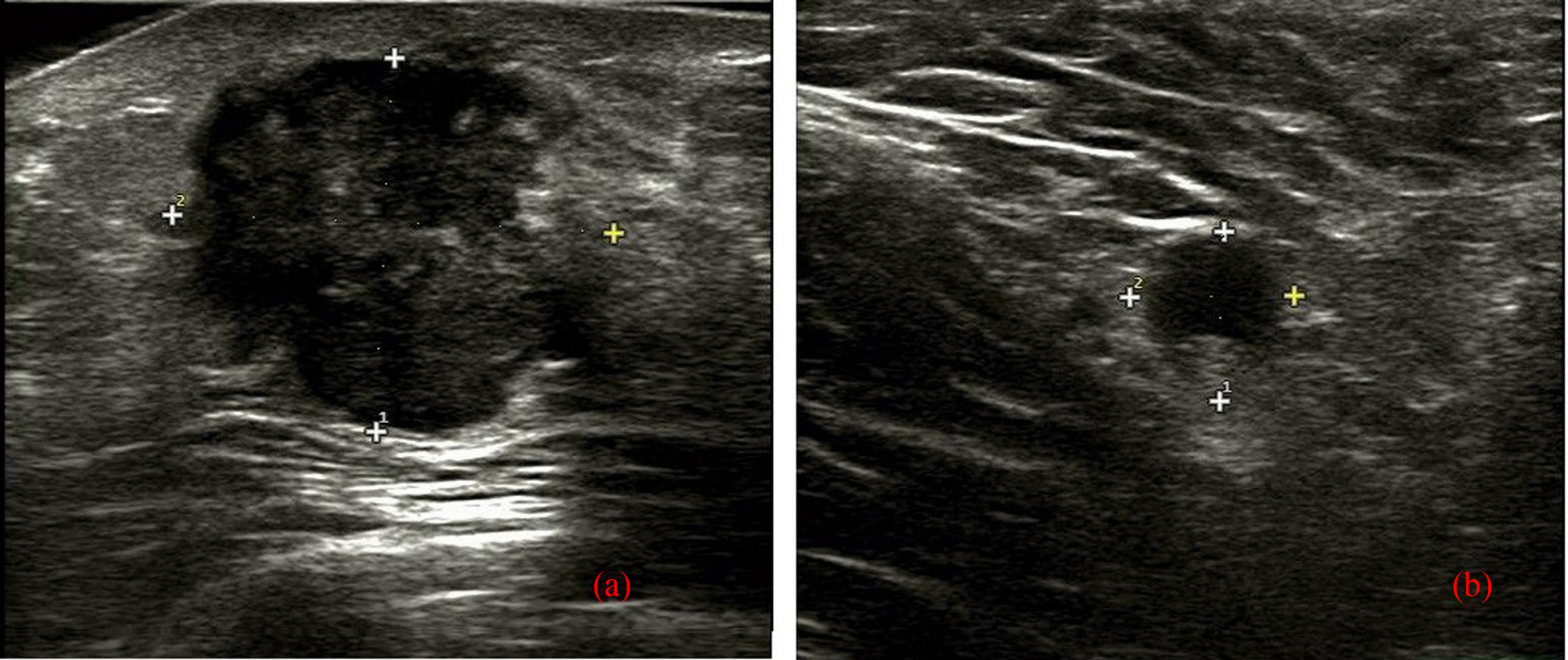
US of MBC with osseous differentiation. US revealed that there was an uneven hypoechoic mass at 9 o’clock near the nipple, with unclear boundary, irregular shape, and posterior acoustic enhancement, and few blood flow signals inside, but without definite calcifications (a) and the suspected enlarged lymph node with a slightly thickened cortical area at the right axillary region (b).

A 52-year-old female was admitted to our hospital with a diagnosis of MBC with osseous differentiation, based on the pathology of the excisional breast mass, without any history of carcinoma or family history of breast cancer. Three months prior, she noticed a mass in her right breast. Physical examination showed a firm, tender mass, approximately 2.5 cm size in the lower outer quarter of the right breast, without a clear boundary. No redness, swelling, pigmentation, or orange peel changes were found on the skin. No invagination, bleeding, or effusion were found in the bilateral nipple. The left breast and the bilateral axillary and supraclavicular lymph nodes were unremarkable. No obvious abnormalities were found in laboratory tests. Breast ultrasound (US) (Figure 1) showed an uneven hypoechoic mass at 9 o’clock near the nipple, and the maximum size was approximately 3.0 cm, with unclear boundary, irregular shape, posterior acoustic enhancement, and few blood flow signals inside. The mass was considered Breast Imaging Reporting and Data System (BI-RADS) category 4A, and a suspected enlarged lymph node with a slightly thickened cortical area at the right axillary region was also indicated by US.

**Figure 2:**
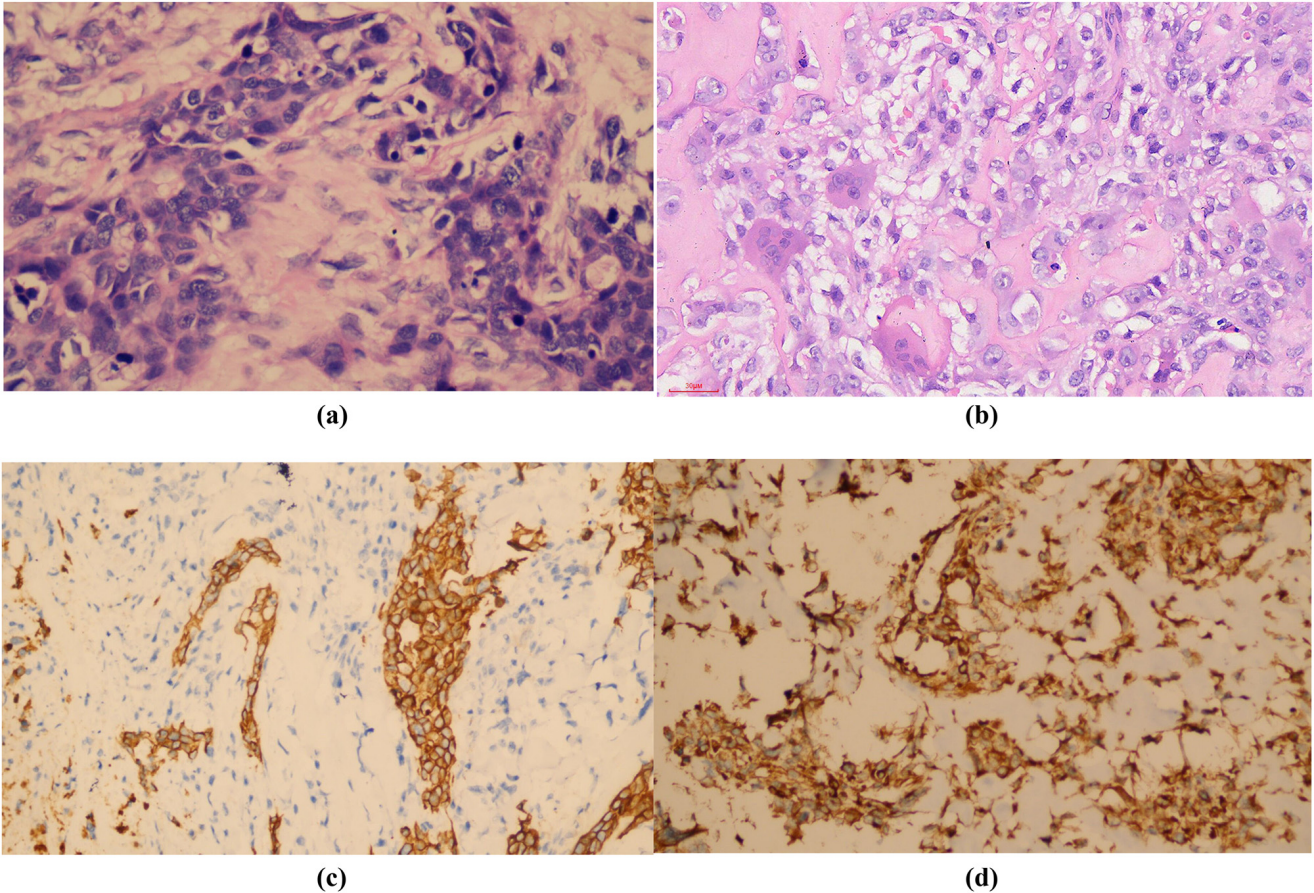
Pathological pictures of MBC with osseous differentiation, excision specimen. Images (a) and (b) showed components of typical IDC (a [20×]) and osteosarcoma (b [20×]). Images (c [10×]) and (d [20×]) showed immunohistochemistry of CK-P and vimentin staining, respectively; both were positive.

**Figure 3:**
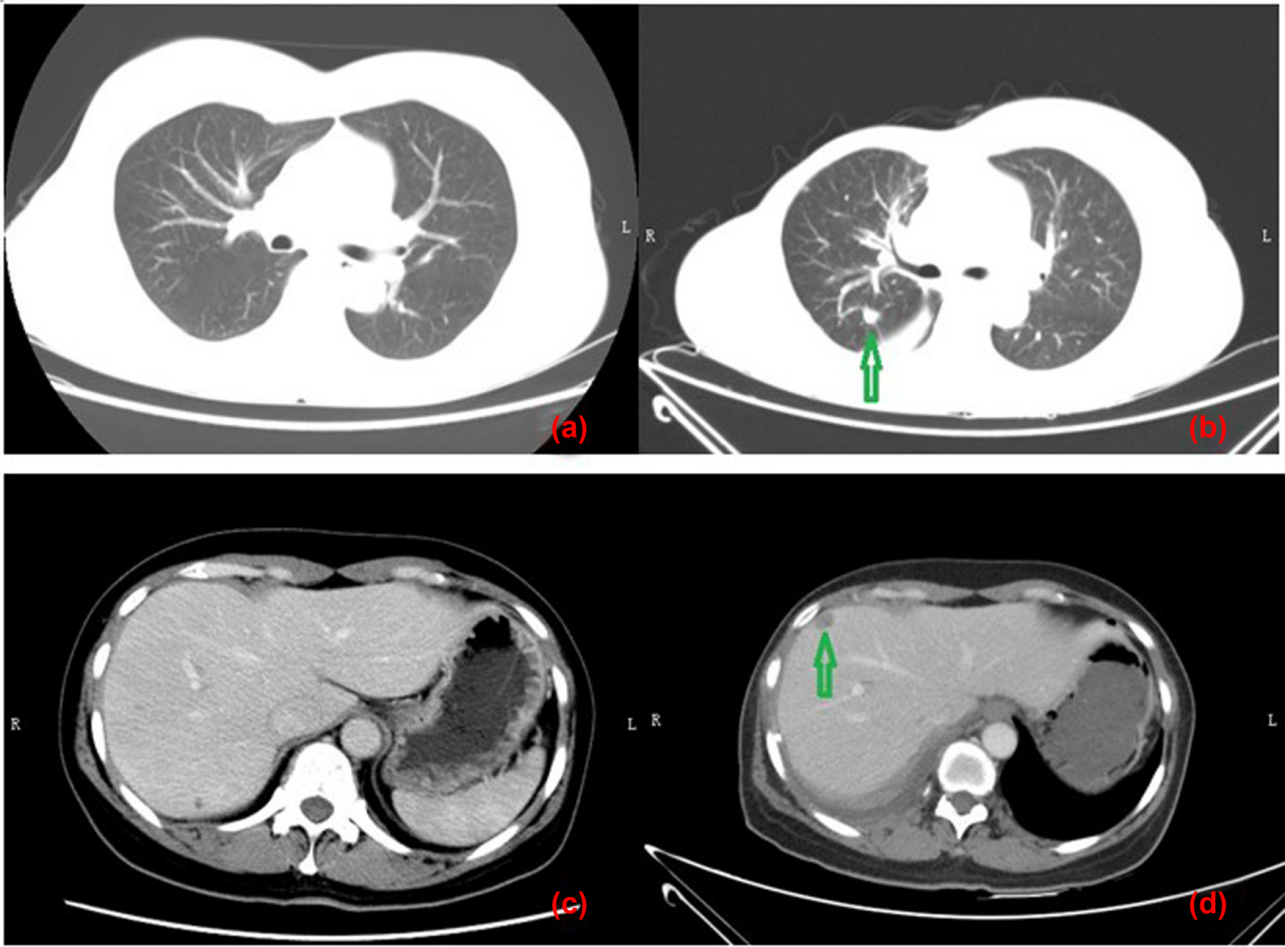
Comparing CT plans of the lung and liver before the modified radical mastectomy and 24 months after the surgery. Images (a) and (c) were CT plans of the lung and liver before the modified radical mastectomy, respectively, and revealed no metastases. Images (b) and (d) were the corresponding plans 24 months after the surgery, and metastases were indicated by the green arrows.

The patient underwent the breast mass excision surgery at that hospital without any further examinations and histopathologically (Figure 2) diagnosed with MBC with osseous differentiation, because there were two different components in the tumor; one was typical invasive ductal carcinoma (IDC) and the other was presenting as osteosarcoma with lots of immature trabeculae, and atypia osteocytes among them; there was no spindle cell component. Immunohistochemical analysis revealed that cells were CD10, CK-P and vimentin positive, estrogen receptor (ER), progesterone receptor (PR), human epidermal growth factor receptor 2 (HER-2), CD34, GATA-3, HMB45, smooth muscle actin (SMA), and p63 negative; proliferative index with KI-67 was 50%. Four days later, she was admitted to our hospital for further treatment. Chest computed tomography (CT) (Figure 3) indicated that there was no metastasis in the lung and liver; emission computed tomography (ECT) findings also revealed no signs of bone metastasis. Considering that the breast mass was a high-grade malignancy, and there was a suspected metastatic lymph node at the right axillary region; doctors suggested a biopsy of the lymph node under the guidance of US. However, the patient and her families refused and requested an excision of the sentinel lymph node. Histological results showed the lymph node metastasized from breast invasive carcinoma. Immunohistochemical analysis revealed that CK5/6 and p63 were positive. Subsequently, a modified radical mastectomy was performed, and there were no other metastatic lymph nodes (0/21). Finally, she was diagnosed with MBC with osseous differentiation, staged as TxN1M0.

**Figure 4:**
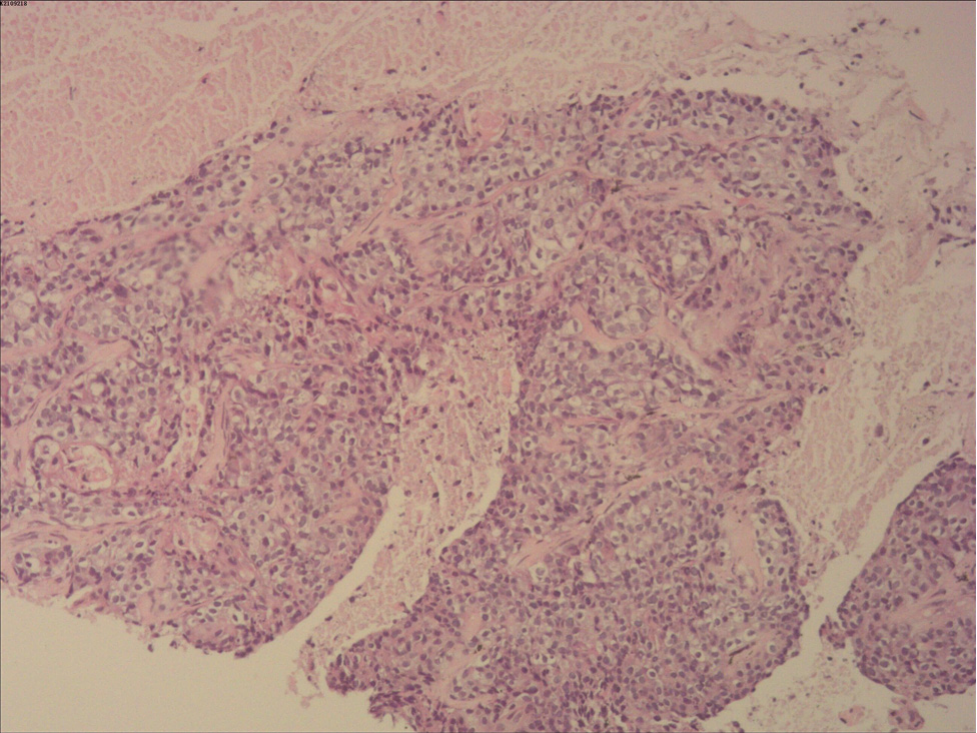
Histopathology of lung nodule (10×). This picture showed that lung nodule was metastasized from breast invasive carcinoma.

The patient had received six cycles of TEC chemotherapy (docetaxel, 700 mg; epirubicin, 100 mg; cyclophosphamide 500 mg; 21 days), initiated after the modified radical mastectomy, and radiotherapy (RT) at a dosage of 48 Gy/25 f/5 days a week to treat the lymph node of the right superior and inferior clavicular fossa and right chest for 5 weeks. The follow-up investigations were advised every 6 months including breast, abdomen and lymph node US, blood routine, liver and kidney function, and tumor markers and chest CT every year. However, she did not perform any checking items for 18 months since RT. Twenty-four months later after surgery, she was admitted again for dyspnea and chest discomfort; chest CT (Figure 3) revealed that there were probable metastatic lesions in lungs (maximum: 29 mm × 27 mm), liver (maximum: 12 mm × 10 mm), and mediastinal lymph nodes (maximum: 26 mm), with pleural effusion in the right thoracic cavity. The lung nodule was diagnosed as metastasis from breast invasive carcinoma by CT guided needle biopsy (Figure 4). Immunohistochemical analysis revealed that cells were ER, PR, PF-L1, SOX10, Napsin A, thyroid transcription factor-1, GCDFP-15 negative, HER-2 (2+) but *in situ* hybridization negative, CK-P and GATA-3 positive, proliferative index with KI-67 was 40%, and BRCA1 was local positive. The patient refused further treatment and died 3 months later. The related indicators of tests and examinations throughout the patient are presented in Table 1.

**Table 1:** The main related indicators throughout the treatment

Parameters time after surgery	Breast mass excision	Modified radical mastectomy	Chemotherapy						RT	Latest hospitalized	Normal reference
(month)	(0)	(0)	1 (0)	2 (1)	3 (2)	4 (3)	5 (4)	6 (5)	(6)	(24)	
HGB (g/L)	NA	140	103	132	132	134	130	134	130	134	115–150
WBC (10^9^/L)	NA	7.42	10.9	7.28	7.30	7.3	5.67	7.79	5.02	6.9	3.5–9.5
CEA (ng/mL)	NA	NA	NA	NA	1.65	NA	NA	0.97	0.97	1.45	0–5
CA153 (U/mL)	NA	NA	NA	NA	7.94	NA	NA	7.24	5.39	14.43	0–25
Breast ultrasound	An uneven hypoechoic mass (sized 3.0 cm) in the right breast a suspected swollen lymph node at the right axillary region	After excision of right breast mass, a suspected swollen lymph node at the right axillary region	NA	NA	(−)	NA	NA	(−)	NA	(−)	
CT	NA	Lung, liver (−)	NA	NA	NA	NA	NA	Lung, liver (−)	NA	**Lung:** scattered nodules (max: 29 mm × 27 mm), pleural effusion; **Liver:** multiple hypo-density nodules (max: 12 mm × 10 mm); **Lymph nodes:** multiple mediastinal lymphadenopathy (max: 26 mm)	
ECT	NA	(−)	NA	NA	NA	NA	NA	NA	NA	NA	

Meanings of some important symbols: (−): normal; NA: this examination was not performed. Abbreviations: HGB: hemoglobin; WBC: white blood cell; CEA: carcinoembryonic antigen; CA153: carbohydrate antigen 153; CT: computed tomography; ECT: emission computed tomography; RT: radiotherapy.


**Informed consent:** Informed consent has been obtained from all individuals included in this study.
**Ethical approval:** The research related to human use has been complied with all the relevant national regulations, institutional policies, and in accordance with the tenets of the Helsinki Declaration, and has been approved by the authors’ institutional review board or equivalent committee.

## 3 Discussion

Metaplastic matrix-producing carcinoma is an aggressive and rare kind of MBC, which contains invasive carcinoma displaying a transition to a cartilaginous and/or osseous stromal matrix lacking an intervening spindle cell component [5,6]. Although there are many published case series of MBC, MBC with osseous differentiation is still rare [7]. A study from India showed that 13 (2.5%) out of total 510 MBC specimens were mesenchymal differentiation, but none was osseous differentiation [8]. The same results were also found in articles including 14 Australian and 38 Turk MBC patients [9,10]. Zhang et al. reported six cases of matrix-producing MBC, but all were chondroid differentiation [11]. The histogenesis and origin of MBC are controversial [4]. The most common hypotheses regarding the histogenesis of MBC are as follows: (1) collision hypothesis, which suggests that carcinomatous and sarcomatous tissues are derived from different progenitor cells [12]; (2) monoclonal hypothesis, which suggests that the components of MBC are monoclonal, and thereby derived from a common multipotent progenitor cell [4]; and (3) epithelial–mesenchymal transition, which suggests that sarcomatous components are derived from the carcinomatous component, or myoepithelial cells due to the positivity of myoepithelial markers including CD10, p63, and SMA [13].

It is reported that MBC mostly occurs in patients aged over 50 years [14] and is characterized by rapid growth generally >2 cm [12], so was our case. The diagnosis of MBC is very difficult, because of no specificity in clinical manifestation and auxiliary examinations. Although calcifications might be detected by auxiliary examinations if there were osteosarcoma components [15,16], it is still difficult to distinguish from breast invasive carcinoma or benign neoplasms including fibroadenoma [17,18,19]. The pathological diagnosis of MBC requires the demonstration of epithelial and heterologous (chondroid or osseous), as well as the absence of spindle cell differentiation. Therefore, it can only be done on a surgical specimen [20].

According to National Comprehensive Cancer Network (NCCN) guidelines of breast cancer screening and diagnosis [21], BI-RADS 4A breast masses should be performed for further examinations or biopsy. Although this case was accurately diagnosed with MBC with osseous differentiation through surgical specimens at the beginning, we still recommend biopsies of the mass and suspicious metastatic lymph node before surgery. Comprehensive preoperative relevant examinations including chest CT, mammogram, magnetic resonance imaging, and emission computed tomography are crucial for clinicians to stage the MBC patient, make a therapeutic regimen, and evaluate the progression and prognosis. The lack of comprehensive preoperative examinations including mammogram of the mass is a limitation of this case report. It has been reported that ER, PR, and HER-2 are negative in more than 90% of MBC, which is similar to the biological characteristics of triple-negative breast cancer (TNBC) [22], while at least one of keratin, including AE1/3, CK7, CK5, CK14, and CK15, is positive. Vimentin, p63, and SMA are other potentially positive markers [14,23]. Findings of our case were consistent with this, and the presence of epithelial components of positive CK-P staining differentiated it from the primary osteosarcoma.

Although the optimal management for this rare malignancy remains unclear, total mastectomy with surgical axillary staging is necessary for the follow-up treatment [24,25,26]. Currently, surgical management guidelines of MBC parallel the recommendation for IDC [27]. Breast-conserving surgery could be performed in patients with early-stage MBC; however, it is reported that modified radical mastectomy is often the preferred option for definitive surgical intervention due to larger tumor size, higher tumor grade, and hormone receptor negativity compared with IDC [28,29,30]. Axillary management is still important. It is reported that MBC patients with positive lymph nodes are associated with poor prognosis [21,31], although only 15–20% of MBC presents axillary nodal metastases [32], less frequently than hematogenous metastasis [12,33]. Therefore, a positive biopsy is recommended when US indicates a suspicious metastatic lymph node. In our case, a modified radical mastectomy was performed, with one axillary node metastasis in total (1/22).

Systemic therapy and/or RT have been received as components of the definitive treatment in most MBC patients, due to the negative prognosis associated with the histology and the increased likelihood of advanced-stage disease at presentation, although the optimal regimen remains undefined [25,34]. Studies have demonstrated variable responses to systemic therapy, it is reported that some MBC cases responded poorly to systemic chemotherapy and had a poor prognosis [35], while some [12,29,36] demonstrated better outcomes with chemotherapy, but no association between RT and prognosis of MBC. However, many studies have reported that postoperative RT is independently associated with improved overall survival and disease-specific survival in MBC patients [37,38,39,40]. In addition, a study of Polamraju et al. revealed that adjuvant chemotherapy and RT improved the prognosis of MBC patients [41]. Considering that the patient we reported was ER- and HER-2-negative MBC with 3.0 cm in size and one positive axillary node, according to the NCCN guidelines [24] and Chinese Society of Clinical Oncology guidelines [42], chemotherapy scheme of TEC and RT to infraclavicular region, supraclavicular area, and any part of the axillary bed at risk were sequentially performed.

The prognosis of MBC is poor [25]. A study comparing outcomes of 5,142 MBC and 50,705 TNBC patients suggested that the 5-year overall survival rate of MBC was 55%, which was less than that of TNBC with an overall survival rate of 72% [41]. It has been reported that approximately 50% of MBC patients present distant metastases after the primary surgery, with lungs and brain being the organs most commonly involved [39,43]. Similarly, the patient in our case was diagnosed with multiple organ metastases from breast invasive carcinoma 24 months later after surgery, including the liver and lungs, and died 3 months later.

For this case, first, it was a rare malignancy with ER, PR, and HER-2 negativity, and high proliferative index with Ki-67 (50%), and 3.0 cm in size and positive axillary lymph node; all the indicators implicated a poor prognosis. Secondly, the patient was advised to review breast carcinoma-related items every 6 months. However, she did not perform any checking items for 18 months since RT and admitted again for dyspnea and chest discomfort, and chest CT revealed scattered metastases to lungs, liver, and mediastinal lymph nodes. It is suggested that regular review plays a crucial role in early detecting metastases; simultaneously, active lifestyle, healthy diet, and timely intervention are important for improving prognosis.

## 4 Conclusion

MBC with osseous differentiation is an extremely rare type of MBC, with a prognosis poorer than that of TNBC. The origin of this disease remains unclear. Auxiliary examinations cannot provide specific information, and a final diagnosis is based on meticulous pathological and comprehensive immunohistochemical examinations. It is still controversial in operation selection, management of axillary lymph nodes, the role of adjuvant chemoradiotherapy, and RT. Further researches regarding the origin, imaging findings, and effective treatment methods are required to improve prognosis.
